# A_2A_ Receptor Activation Restores Lipid and Mitochondrial Homeostasis, Limiting *Mycobacterium leprae* Persistence in Human Monocytes

**DOI:** 10.3390/metabo16050304

**Published:** 2026-04-29

**Authors:** Antonio M. Rodrigues Pereira, Plinio M. Freire dos Santos, Thabatta L. S. A. Rosa, Chyntia Díaz Acosta, Karina G. C. Vasconcelos, Luisa D. Gutierres, Fabrício M. R. Costa, Leticia M. S. Lery, Rafael Garrett, Marina A. Alves, André A. Dias, Flavio A. Lara, Luciana Silva Rodrigues, Roberta Olmo Pinheiro, Maria Cristina V. Pessolani, Márcia Berrêdo-Pinho

**Affiliations:** 1 Laboratory of Cellular Microbiology, Oswaldo Cruz Institute, Oswaldo Cruz Foundation (FIOCRUZ), Rio de Janeiro 21040-900, Brazil; rj.antoniomarcos@gmail.com (A.M.R.P.); pliniosantos94@gmail.com (P.M.F.d.S.); thab.landrezo@gmail.com (T.L.S.A.R.); aadias2005@yahoo.com.br (A.A.D.); flavioalveslara2000@gmail.com (F.A.L.); cpessola@gmail.com (M.C.V.P.); 2 Department of Molecular Biology and Biotechnology, Health Sciences Research Institute (IICS), National University of Asunción, Asunción 111421, Paraguay; 3 Center for Integrative Biology, Faculty of Sciences, Universidad Mayor, Santiago 8580745, Chile; karina_girardi@hotmail.com; 4 Gerosciense Center for Brain Health and Metabolism (GERO), Santiago 8580745, Chile; 5 Laboratory of Immunopathology, Medical Sciences Faculty, Rio de Janeiro State University (FCM/UERJ), Rio de Janeiro 20550-900, Brazil; fabricio.mrc@gmail.com (F.M.R.C.); lrodrigues.uerj@gmail.com (L.S.R.); 6 Metabolomics Laboratory, Institute of Chemistry, Federal University of Rio de Janeiro (UFRJ), Rio de Janeiro 21941-909, Brazil; rafael_garrett@iq.ufrj.br; 7 Laboratory of Metabolomics Applied to Systems Medicine, Natural Products Research Institute, Federal University of Rio de Janeiro (UFRJ), Rio de Janeiro 21941-909, Brazil; marina.amaral@iq.ufrj.br; 8 Leprosy Laboratory, Oswaldo Cruz Institute, Oswaldo Cruz Foundation (FIOCRUZ), Rio de Janeiro 21040-900, Brazil; robertaolmo@gmail.com

**Keywords:** leprosy, host–pathogen interactions, adenosine receptor A_2A_, lipid droplets, mitochondrial function, *Mycobacterium leprae*

## Abstract

**Background/Objectives:** Leprosy is a chronic infection caused by *Mycobacterium leprae* that, in addition to Schwann cells, macrophages, and adipocytes, also infects human peripheral blood monocytes and subverts their metabolism in its favor. Infection is marked by cholesterol and fatty acid accumulation in lipid droplets (LDs), and a reduction in mitochondrial membrane potential (Δψm). Previous studies showed that *M. leprae* downregulates adenosine receptor A_2A_ (A_2A_R) expression in Schwann cells, while activation reduces LD accumulation and bacterial viability. Since A_2A_R controls immunometabolic response, we investigated whether A_2A_R signaling restrains *M. leprae*-driven reprogramming in monocytes. **Methods:** Peripheral blood mononuclear cells from healthy donors were enriched for monocytes and infected with *M. leprae* in the presence or absence of adenosinergic modulators (5′AMP, adenosine (ADO), A_2A_R agonist CGS21680, the antagonist ZM241385, or A_2B_R antagonist, MRS1754). We used flow cytometry, fluorescence microscopy, and RT-qPCR to evaluate purinergic components expression and bacillary viability. LDs and Δψm were measured by fluorescence microscopy, and extracellular levels of inosine (INO) and hypoxanthine (HPX) by LC-MS/MS. **Results:** The results show that infection increased CD39, ADA, A_2A_R and A_3_R expression, decreased ENT1, A_1_R and A_2B_R, and raised extracellular INO and HPX. In addition, 5′AMP, ADO and CGS21680 reversed infection-induced LD accumulation. CGS21680 also restored Δψm and decreased intracellular *M. leprae* viability. **Conclusions:** Our data suggest that *M. leprae* suppresses A_2A_R signaling to favor its survival in monocytes, indicating that the extracellular ADO–A_2A_R pathway may be a potential target to limit early *M. leprae* infection.

## 1. Introduction

Leprosy is a chronic infectious disease caused by *Mycobacterium leprae* and *Mycobacterium lepromatosis* [1]. Each year, over 180,000 new cases occur, mainly in India, Brazil, and Indonesia [2]. In the Americas, Brazil accounts for about 90% of all new cases, reflecting its endemic nature, and the persistence of pediatric cases indicates ongoing community transmission [2]. Leprosy primarily affects skin macrophages and peripheral nerves, leading to chronic inflammation, loss of sensation, muscle weakness, and, if untreated, permanent disabilities [3,4]. Despite advances in diagnosis and treatment, it remains a stigmatized condition that profoundly affects patients’ social participation and quality of life [5].

Although multidrug therapy (MDT) is effective, its prolonged duration remains a major limitation in leprosy control. In 2021, the World Health Organization (WHO) presented the Global Leprosy Strategy 2021–2030 [6], which points out ways to eliminate leprosy as a public health problem. One of the strategies focuses on developing therapies that complement MDT to overcome the challenges posed by drug-resistant strains, prolonged treatment durations, and side effects that can compromise patient adherence and quality of life [6]. *M. leprae* preferentially infects Schwann cells and skin macrophages [3,4]. More recently, it has also been described to infect and persist inside adipocytes [7]. In addition, monocytes are rapidly recruited to infection sites, peripheral nerves, or subcutaneous white adipose tissue [8,9,10]. Their cellular functions and metabolic processes can be subverted by *M. leprae* to promote persistence and establish the infection [11,12,13]. For example, *M. leprae* can inhibit the polarization of monocytes from healthy donors into an M1 macrophage profile. This reduces the secretion of pro-inflammatory cytokines such as IL-6, IL-1β, IL-12p70, and TNF-α, and lowers expression of the M1 markers CCR7 and CD40 [11,12]. It also downregulates the mitochondrial function (Δψm), limits fatty acid oxidation, and contributes to the foamy phenotype induced by infection [13]. Monocyte immunometabolic dysfunction has also been reported in *M. tuberculosis* and *P. falciparum* infections, where lipid remodeling, hemozoin-induced oxidative stress, and lipid peroxidation impair antimicrobial functions [14,15]. Thus, human monocytes provide a relevant model system for dissecting early host–pathogen interactions and identifying host-directed strategies for disease control.

A hallmark of *M. leprae* infection is its ability to subvert the host cell’s lipid metabolism, inducing the formation of lipid droplets (LDs) [16,17,18,19,20], organelles rich in cholesteryl esters and triacylglycerols [19,20], which serve as reservoirs for neutral lipids, playing a vital role in maintaining cellular energy balance, contributing to the evasion mechanisms employed during mycobacterial infection [21]. Our group has demonstrated, through both *in vitro* and *in vivo* studies, that treatment with cholesterol biosynthesis inhibitors, such as statins, can reduce LD accumulation and, consequently, intracellular viability of *M. leprae* [20,22]. Recently, we strengthened this finding by showing that pamidronate, another inhibitor of the mevalonate pathway, also decreased the viability of *M. leprae* in infected macrophages [23], underscoring the importance of LDs in the persistence of the bacillus. We also observed that *M. leprae* downregulates adenosine A_2A_ receptor (A_2A_R) expression in Schwann cells. However, despite this downregulation, activation of this receptor with the selective agonist CGS21680 reduced LD formation in *M. leprae*-infected Schwann cells, coinciding with decreased intracellular bacillary viability [24].

A_2A_R is part of the purinergic system, a complex cellular communication network first proposed by Geoffrey Burnstock in 1972 [25]. It consists of extracellular ligands, including nucleotides/nucleosides (e.g., ATP, adenosine (ADO), and inosine (INO); cell-surface receptors, classified into P1 and P2 receptors; and ectoenzymes that control extracellular ligand levels [25]. The P1 receptors are subdivided into A_1_, A_2A_, A_2B,_ and A_3_. A_2A_R and A_2B_R are coupled to the stimulatory G protein. In contrast, A_1_R and A_3_R are coupled to the inhibitory G protein [26,27]. Although the main ligand of these receptors is ADO, recent studies show that INO can activate some of these receptors, including A_2A_R [28]. The P2 receptors are classified into two families, P2X and P2Y, and bind nucleotides, such as extracellular ATP [27]. Through these elements, purinergic signaling regulates many cellular functions, such as inflammatory responses, immune activation, and pathogen clearance [29,30]. Among the ectoenzymes of the purinergic system, CD39, also known as ecto-nucleoside triphosphate diphosphohydrolase-1 (ENTPD1), that hydrolysis extracellular ATP and adenosine diphosphate (ADP) into extracellular 5′adenosine monophosphate (5′AMP) [28]. Another ectoenzyme that regulates extracellular nucleotide levels is ecto-5′-nucleotidase CD73 (NT5′E). This enzyme hydrolyzes the extracellular 5′AMP to generate extracellular ADO [31]. Both enzymes are expressed in different cell types, such as immune cells and endothelial cells, and both CD39 and CD73 participate in killing invading pathogens [32,33]. Interestingly, CD39 has been identified as a regulator of the pro-inflammatory response [34], whereas CD73 contributes to lipid metabolism regulation and supports host–pathogen adaptation mechanisms that enhance intracellular persistence of bacterial pathogens [35,36,37].

ADO is an immunomodulatory molecule [38,39,40] that, in the extracellular microenvironment, regulates lipolysis, cholesterol efflux, and LD formation, suggesting its involvement in lipid metabolism and energy homeostasis [41,42,43,44,45]. The levels of extracellular ADO are regulated by adenosine deaminase (ADA), which deaminates ADO into inosine (INO) [46]. In leprosy, elevated ADA activity, particularly during reactional episodes, supports the notion that purine metabolism is dynamically regulated during disease progression [47,48]. INO extracellular levels can be regulated by ectoenzyme purine nucleoside phosphorylase (ecto-PNP), which metabolizes INO into hypoxanthine (HPX) [49]. Further, adenosine transporters, such as equilibrative nucleoside transporters (ENTs), including ENT1, mediate the reuptake of ADO, INO, and HPX into cells, thereby regulating their extracellular concentrations [50]. The regulation of extracellular ADO levels has been shown to dampen inflammation, promote an immunosuppressive microenvironment, and aid the establishment of infections by *Mycobacterium tuberculosis*, *Leishmania* spp., *Toxoplasma gondii*, *Streptococcus pneumoniae*, Salmonella, and *Plasmodium* sp. [30,51,52]. In this context, activation of the A_2A_R has been associated with the regulation of host cell responses, contributing to the elimination of *Candida albicans* and *M. leprae* [24,53]. Furthermore, A_2A_R activation has been associated with reduced intracellular cholesterol levels, a mitoprotective effect, and an increase in Δψm [43,44,45].

In this study, we aimed to investigate how *M. leprae* regulates purinergic signaling in monocytes and how this influences host metabolic reprogramming and bacterial persistence. Our data showed that *M. leprae* infection increased the expression of the ectoenzymes CD39, CD73, and ADA, leading to increased extracellular levels of INO and HPX. Infection also increased A_2A_R expression, and its activation reversed LD accumulation and decreased the intracellular viability of *M. leprae*. Furthermore, we identify A_2A_R involvement in reversing the Δψm downregulation induced by *M. leprae* infection. These findings provide new insights into the pathogenesis of leprosy and suggest that the A_2A_R is a promising target for complementary therapeutic strategies.

## 2. Materials and Methods

### 2.1. Mycobacterium Leprae

Live *Mycobacterium leprae* (Thai-53 strain; taxid:1769) was kindly provided by Dr. Patrícia Sammarco Rosa (Lauro de Souza Lima Institute, Bauru, SP, Brazil). The bacilli were maintained and harvested from the footpad of athymic nude mice (BALB/c nu/nu; RRID: IMSR_JAX:002019) as described by Shepard [54], under protocol number ILSL003/21 approved by the Institutional Animal Care and Use Committee (CEUA) of the Lauro de Souza Lima Institute. All animal procedures were conducted and reported in accordance with the ARRIVE guidelines for reporting animal research.

For isolation, the bacilli were quantified by optical microscopy using the Shepard and McRae method [55]. Bacterial viability was determined using the LIVE/DEAD™ BacLight™ Bacterial Viability Kit (Invitrogen, Thermo Fisher Scientific, Waltham, MA, USA; Cat# L7007) according to the manufacturer’s instructions; only suspensions with a minimum viability of 90% were utilized for infection assays. To generate killed bacteria, a subset of the bacilli was subjected to lethal gamma irradiation (104 Gy) [56] at a specialized facility (Acelétron, Rio de Janeiro, Brazil). The absence of viability in irradiated samples was confirmed by Live/Dead.

### 2.2. Isolation and Enrichment of Primary Human Monocytes

The use of human peripheral blood samples was approved by the Research Ethics Committee of the Oswaldo Cruz Institute, Oswaldo Cruz Foundation (CEP FIOCRUZ/IOC), under approval number 1.538.467 (CAAE: 55367216.0.0000.5248), and performed in compliance with the principles of the Declaration of Helsinki.

Human peripheral blood mononuclear cells (PBMCs) were obtained from buffy coats of healthy adult donors of both sexes aged 21 to 46 years. Inclusion criteria comprised healthy adult donors with no reported history of infectious, inflammatory, or autoimmune diseases. Exclusion criteria included ongoing infections, chronic inflammatory conditions, or recent immunomodulatory treatments. In all experiments, buffy coats from at least three independent donors were used, and all assays were performed in technical triplicate.

Blood was collected in sodium heparinized tubes (BD Vacutainer^®^; BD Biosciences, San Jose, CA, USA; Cat# 367874) and separated by density gradient centrifugation using Ficoll-Paque PREMIUM (Cytiva, Marlborough, MA, USA; Cat# 17544202), as previously described [45]. Briefly, whole blood was diluted 1:1 with PBS (pH 7.4, without Ca^2+^ and Mg^2+^), carefully layered onto the density gradient medium, and centrifuged at 492× *g* for 30 min at room temperature with the brake off. The mononuclear cell layer at the interface was collected and subjected to two wash cycles in cold PBS (300× *g*, 10 min).

Cell viability was confirmed to be >95% using the trypan blue exclusion method. For monocyte enrichment via plastic adherence, PBMCs were seeded at a density of 2 × 10^6^ cells/mL in RPMI 1640 (Gibco, Thermo Fisher Scientific; Cat# 11875093) medium supplemented with 2% fetal bovine serum (FBS) (Gibco, Thermo Fisher Scientific; Cat# 12483020). Following a 2 h incubation at 37 °C in a 5% CO_2_ atmosphere, non-adherent cells were removed by three vigorous washes with warm PBS. The resulting adherent monocyte-enriched population was then maintained in fresh RPMI 1640 supplemented with 10% FBS for subsequent experiments. A purity of 98% was obtained, which refers specifically to the final gated population used for flow cytometry analysis, rather than to the overall monocyte proportion within the total PBMC culture.

### 2.3. Flow Cytometry Analysis

PBMCs were obtained from independent healthy donors, seeded at approximately 2 × 10^6^ cells per well in 24-well culture plates, enriched for monocytes by plastic adherence, and cultured in RPMI 1640 medium supplemented with 10% FBS. Monocytes were infected or not with *M. leprae* at a multiplicity of infection (MOI) of 10:1 for 48 h at 33 °C in a humidified atmosphere containing 5% CO_2_, using RPMI 1640 medium supplemented with 10% FBS. Following infection, cells were harvested, washed with PBS, and incubated with a blocking solution containing 10% human AB serum in PBS for 30 min at 4 °C to reduce nonspecific antibody binding.

Cells were then stained with mouse anti-human CD14 antibody conjugated to PerCP-Cy5.5 (Thermo Fisher Scientific, Cat# 15-0149-42, RRID: AB_2573058) to identify monocytes. Surface expression of purinergic receptors and ectoenzymes was assessed using the following primary antibodies diluted 1:250 in blocking solution for 30 min at 4 °C: rabbit anti-A_2A_R antibody (Alomone Labs, Jerusalem, Israel Cat# AAR-002, RRID: AB_2039707), rabbit anti-A_1_R antibody (Alomone Labs Cat# AAR-006, RRID: AB_2039705, rabbit anti-A_2B_R antibody (Alomone Labs Cat# AAR-003; RRID: AB_2039709), rabbit anti-A_3_R antibody (Alomone Labs Cat# AAR-004; RRID: AB_2039711), mouse anti-CD39 antibody (Abcam, Cambridge, UK; Cat# ab30422, RRID: AB_2100068), mouse anti-CD73 antibody (Abcam Cat# ab54217, RRID: AB_879692), and rabbit anti-adenosine deaminase (ADA) antibody (Santa Cruz Biotechnology, Dallas, TX, USA; Cat# sc-25747, RRID:AB_2273599).

At the end of the incubation period, cells were washed with PBS and incubated with species-appropriate secondary antibodies conjugated to Alexa Fluor 488: goat anti-rabbit IgG antibody (Thermo Fisher Scientific Cat# A11034, RRID: AB_2576217) or goat anti-mouse IgG antibody (Thermo Fisher Scientific Cat# A11001, RRID: AB_2534069), diluted 1:500 for 30 min at 4 °C in the dark. Cells were subsequently fixed in 1% paraformaldehyde for 30 min, washed twice with PBS, and resuspended in PBS for acquisition, according to a protocol adapted from Díaz-Acosta et al. [16].

Data were acquired on a BD FACSCanto™ II flow cytometer (BD Biosciences, San Jose, CA, USA) using BD FACSDiva software v6.1.3, with acquisition of at least 10,000 CD14^+^ events per sample. Compensation was performed using single-stained controls, and appropriate isotype-matched controls were included for all antibodies. Data analysis was carried out using FlowJo software v10 (RRID: SCR_008520). Gating strategies were defined based on forward and side-scatter parameters to exclude debris and doublets, followed by selection of CD14^+^ monocytes. Median fluorescence intensity (MFI) values were used for quantitative comparisons. Analyses were performed using biological replicates derived from independent donors. During flow cytometry acquisition, photomultiplier tube (PMT) voltages were adjusted to clearly resolve negative and positive populations on a logarithmic scale. As a result, in one analysis, the unstained control peak appears around 10^2^ rather than 10^0^. However, all samples were acquired using identical instrument settings, ensuring consistent comparison among experimental groups.

### 2.4. Fluorescence Microscopy Analysis

PBMCs were obtained from independent healthy donors and seeded at approximately 2 × 10^6^ cells per well onto sterile glass coverslips in 24-well culture plates. After the adherence period, non-adherent cells were removed, resulting in an enriched monocyte population for subsequent analyses. Adherent monocytes were infected or not with live *M. leprae* at a multiplicity of infection (MOI) of 10:1 and incubated for 48 h at 33 °C in a humidified atmosphere containing 5% CO_2_.

Following infection, cells were fixed with 4% paraformaldehyde (PFA) for 20 min at 4 °C and washed twice with PBS. Fixed cells were permeabilized and blocked using a solution containing 0.1% Triton X-100 (Sigma-Aldrich, St. Louis, MO, USA; Cat# HFH10) and 1% bovine serum albumin (BSA; Sigma-Aldrich, Cat# A2153) in PBS for 30 min at room temperature to reduce nonspecific antibody binding, following a protocol adapted from Díaz-Acosta et al. [16]. Cells were then incubated overnight at 4 °C with the following primary antibodies diluted 1:300 in blocking solution: rabbit anti-A_2A_R antibody (Alomone Labs Cat# AAR-002; RRID: AB_2039707), and rabbit anti-ENT1 antibody (Abcam Cat# ab182023; RRID: AB_2732846).

After primary antibody incubation, cells were washed twice with PBS and incubated for 2 h at room temperature in the dark with species-appropriate secondary antibodies diluted 1:500 in blocking solution: Alexa Fluor 488-conjugated goat anti-rabbit IgG (Thermo Fisher ScientificCat# A11034; RRID: AB_2576217) for A_2A_R detection, or Alexa Fluor 594-conjugated goat anti-rabbit IgG (Thermo Fisher ScientificCat# A-11012, RRID: AB_2534079) for ENT1 detection. Cell nuclei were counterstained with DAPI (1 µg/mL; Thermo Fisher Scientific, Waltham, MA, USA; Cat# D1306) for 5 min at room temperature, followed by two PBS washes. Coverslips were mounted onto glass slides using ProLong™ Diamond Antifade Mountant (Thermo Fisher Scientific, Waltham, MA, USA; Cat# P36961).

Fluorescence images were acquired using an Axio Observer Z1 epifluorescence microscope (Carl Zeiss, Oberkochen, Germany) equipped with a 40× objective. Image acquisition settings (exposure time, gain, and illumination intensity) were kept constant across all experimental conditions. For each condition, at least 20 randomly selected fields were imaged, corresponding to approximately 200 cells per condition, which were considered technical replicates. Image acquisition and quantitative analysis were performed in a blinded manner whenever applicable.

Quantification of fluorescence intensity was performed using ImageJ software (NIH, Bethesda, MD, USA; version 1.52a) (RRID: SCR_003070). MFI was measured for individual cells after background subtraction and normalized to cell number based on nuclear DAPI staining. Data from independent donors were pooled for statistical analysis.

### 2.5. Lipid Droplet Staining and Quantification

PBMCs were obtained from independent healthy donors and seeded at approximately 2 × 10^6^ cells per well onto sterile glass coverslips in 24-well culture plates. After the adherence period, non-adherent cells were removed, resulting in an enriched monocyte population for subsequent analyses. The cells were cultured in RPMI 1640 medium supplemented with 2% FBS throughout the experimental period. Monocytes were infected or not with live *M. leprae* at a MOI of 10:1 and incubated for 48 h at 33 °C in a humidified atmosphere containing 5% CO_2_.

Cells were pretreated for 30 min with the following adenosinergic modulators: adenosine (ADO; 100 µM; Sigma-Aldrich, Cat# A9251), 5′-adenosine monophosphate (5′-AMP; 100 µM; Sigma-Aldrich, Cat# A1752) the selective adenosine A_2A_R agonist CGS21680 (50 nM or 100 µM; Tocris Bioscience, Shanghai, China, Cat# 1063), the A_2A_R antagonist ZM241385 (50 nM; Sigma-Aldrich, Cat#Z0153), the adenosine A_2B_R antagonist MRS1754 (50 nM; Sigma-Aldrich, Cat# M6316), or the CD73 ecto-5′-nucleotidase inhibitor AMPCP (5 µM; Sigma-Aldrich, Cat#M8386). Following pretreatment, monocytes were infected or not with live or gamma-irradiated (dead) *M. leprae* at a multiplicity of infection (MOI) of 10:1 for 48 h at 33 °C, as described above.

After infection, cells were fixed with 4% paraformaldehyde (PFA) for 20 min at 4 °C. Intracellular LDs were stained using Oil Red O (Sigma-Aldrich, Cat# O0625) according to established protocol [16]. Cell nuclei were counterstained with DAPI (Invitrogen, Cat# D1306), and coverslips were mounted using ProLong™ Diamond Antifade Mountant (Thermo Fisher Scientific, Waltham, MA, USA; Cat# P36961).

LDs were visualized using an Axio Observer Z1 fluorescence microscope (Carl Zeiss, Oberkochen, Germany) equipped with a 40× objective. For morphometric analysis, images were processed using ImageJ software (RRID: SCR_003070). The relative LD area was quantified by thresholding the Oil Red O signal and normalizing the total lipid area to the number of DAPI-stained nuclei. A minimum of 250 cells per condition were analyzed across three independent biological replicates.

### 2.6. Mitochondrial Membrane Potential (Δψm) Analysis

Adherent monocytes were obtained as previously described. The cells were cultured in RPMI 1640 medium supplemented with 2% FBS throughout the experimental period. Monocytes were infected or not with live *M. leprae* at a MOI of 10:1 and incubated for 48 h at 33 °C in a humidified atmosphere containing 5% CO_2_. Cells were pretreated with CGS21680 (100 µM) or ZM241385 (50 nM) for 30 min, followed by infection, with live *M. leprae* or not, as described above. Δψm was assessed using tetramethylrhodamine methyl ester perchlorate (TMRM; Sigma-Aldrich, St. Louis, MO, USA; Cat# T668), a potentiometric fluorescent dye used in non-quenching mode to allow for relative comparisons of mitochondrial polarization. Cells were incubated with 0.5 nM TMRM for 30 min at 33 °C in the dark. As a positive control for mitochondrial depolarization, the protonophore carbonyl cyanide m-chlorophenyl hydrazone (CCCP; Sigma-Aldrich, St. Louis, MO, USA; Cat# C2759) was added at a final concentration of 15 µM, 10 min prior to TMRM incubation, following the protocol previously published by Medeiros [57].

Following staining, cells were washed with warm phosphate-buffered saline and immediately imaged by fluorescence microscopy using an Axio Observer Z1 microscope (Carl Zeiss, Oberkochen, Germany) equipped with a 40× objective. For each experimental condition, at least 20 randomly selected fields were acquired, corresponding to approximately 200 cells per condition, which were considered technical replicates. Image acquisition parameters were kept constant across all experimental groups.

Quantification of mitochondrial fluorescence intensity was performed using ImageJ software (RRID: SCR_003070). Δψm was expressed as the ratio of TMRM fluorescence intensity in the absence versus presence of CCCP, as previously described, allowing normalization for non-mitochondrial background signal and inter-experimental variability. Image analysis was performed in a blind manner with respect to treatment groups.

### 2.7. M. leprae Viability Assessment by 16S rRNA/DNA Ratio

To evaluate the impact of adenosinergic signaling on intracellular *M. leprae* survival, monocytes were enriched from approximately 10^7^ PBMCs and seeded in 6-well plates by plastic adherence. Cells derived from independent healthy donors were used as biological replicates. Monocytes were pretreated for 30 min with CGS21680 (50 nM or 100 µM), ZM241385 (50 nM), or vehicle control (1% DMSO), followed by infection with live *M. leprae* at MOI 10:1, and cultured as previously described.

Following infection, total RNA was extracted using TRIzol reagent (Thermo Fisher Scientific, Cat# 15596026) according to the manufacturer’s instructions, as described in [16]. RNA concentration and purity were assessed by spectrophotometry based on the A_260_/A_280_ ratio. Complementary DNA (cDNA) was synthesized from 250 ng of total RNA using GoScript™ Reverse Transcriptase with random primers (Promega, Madison, WI, USA; Cat# A5001). In parallel, genomic DNA (gDNA) was extracted and purified from the same samples as previously described by Martinez [58], enabling paired analysis of RNA and DNA derived from the same biological material.

The viability of *M. leprae* was assessed by determining the ratio of 16S ribosomal RNA (cDNA) to 16S DNA (DNA) using quantitative real-time PCR (RT-qPCR), according to Amplification was performed using primers and a TaqMan probe specific for the *M. leprae* 16S rRNA region: forward primer, 5′-GCATGTCTTGTGGTGGAAAGC-3′; reverse primer, 5′-CACCCCACCAACAAGCTGAT-3′; and TaqMan probe, 5′-CATCCTGCACCGCA-3′ (6-FAM/NFQ-MGB).

PCR efficiency for each biological replicate was independently calculated using LinRegPCR software v2020.2.0.1 (RRID: SCR_015465). To exclude genomic DNA contamination, no–reverse transcriptase (−RT) controls were included in all experiments. Bacterial viability was expressed as the relative percentage of the 16S rRNA/DNA ratio normalized to the vehicle-treated control group.

### 2.8. Quantification of Extracellular Nucleosides by Liquid Chromatography–Mass Spectrometry (LC–MS/MS)

Adherent monocytes were obtained as previously described. The cells were cultured in RPMI 1640 medium supplemented with 2% FBS throughout the experimental period. Monocytes were infected or not with live *M. leprae* at a MOI of 10:1 and incubated for 48 h at 33 °C in a humidified atmosphere containing 5% CO_2_. Culture supernatants were collected after 48 h. Samples were clarified by centrifugation and stored at −70 °C until metabolite extraction. For metabolite extraction, 120 µL of cell-free supernatant was mixed with 800 µL of ice-cold methanol containing internal standards (caffeine, 5-fluorouridine, 5-fluorouracil, and p-fluoro-DL-phenylalanine; final concentration 3 µg/mL). Samples were vortexed for 60 s, incubated at 4 °C for 20 min to allow protein precipitation, and centrifuged at 21,000× *g* for 15 min at 10 °C. A total of 780 µL of the supernatant was transferred to a clean tube, evaporated to dryness under a nitrogen stream at 40 °C for 80 min, and stored at −20 °C until analysis. Dried extracts were reconstituted in 50 µL of a 7:3 (*v*/*v*) water: acetonitrile solution prior to LC–MS/MS analysis.

Chromatographic separation was performed using a Waters XBridge BEH Amide column (4.6 × 150 mm, 3.5 µm particle size) maintained at 45 °C. The mobile phase consisted of (A) Milli-Q water containing 10 mM ammonium acetate and 0.05% ammonium hydroxide, and (B) 95% acetonitrile containing 10 mM ammonium acetate and 0.05% ammonium hydroxide with 5% Milli-Q water. A total volume of 6 µL was injected per sample. Gradient elution was carried out at a flow rate of 0.5 mL/min using the following program: 0–2 min, 95% B; 2.1–18 min, 50% B; 18.1–21 min, 50% B; 21.1–28 min, 95% B, resulting in a total run time of 28 min per sample. Mass spectrometric analysis was performed on a TSQ Quantiva™ triple-quadrupole mass spectrometer (Thermo Fisher Scientific, San Jose, CA, USA) equipped with an electrospray ionization (ESI) source operated in negative-ion mode. Instrument parameters were as follows: The spray voltage was set to 2500 V; sheath gas, 10 arbitrary units; auxiliary gas, 21 arbitrary units; sweep gas, 1 arbitrary unit; ion transfer tube temperature, 350 °C; vaporizer temperature, 400 °C. Quantification of ADO, inosine (INO), and hypoxanthine (HPX) was carried out using multiple reaction monitoring (MRM) with the following transitions: ADO, *m*/*z* 266.2 → 133.8; INO, *m*/*z* 266.7 → 134.6; and HPX, *m*/*z* 134.9 → 92.04.

External calibration curves were generated using authentic standards prepared in a 7:3 (*v*/*v*) water:acetonitrile solution over a concentration range of 0.001 to 400 µg/mL, containing the internal standard 5-fluorouridine (3 µg/mL). Quantification was performed by peak integration using TraceFinder software version 4.1 (RRID: SCR_023045). Sample acquisition and data analysis were conducted in a blind manner with respect to experimental conditions. Each experimental group included at least three biological replicates (independent donors) and technical triplicates for LC–MS/MS analysis. Internal standards and quality control samples were used to monitor extraction efficiency and instrument performance throughout the analytical runs.

### 2.9. Cellular Toxicity

To ensure that the observed effects of adenosinergic modulation were not due to drug-induced toxicity, cell viability was assessed using the 3-(4,5-dimethylthiazol-2-yl)-2,5-diphenyltetrazolium bromide (MTT) (Sigma-Aldrich, Cat# M6494) colorimetric assay [59]. Enriched monocytes as described below were seeded at a density of 5 × 10^4^ cells/well in 96-well plates and treated for 48 h with ADO (100 µM), 5′AMP (100 µM), CGS21680 (50 nM), ZM241385 (50 nM), MRS1754 (50 nM), or AMPCP (5 µM).

Cells treated with the vehicle (1% DMSO) served as the negative control, while cells treated with 10% DMSO served as the positive control for cell death. After 48 h of incubation, MTT reagent (final concentration: 0.5 mg/mL) was added to each well and incubated for 4 h at 37 °C. The resulting formazan crystals were solubilized in 100 µL of 10% sodium dodecyl sulfate (SDS) in 0.01N HCl, following the protocol previously published by Diaz Acosta [11]. Absorbance was measured at 590 nm using an EON microplate spectrophotometer controlled by Gen5 Microplate Reader and Imager Software v2.07.10 (RRID: SCR_017317). Cell viability was expressed as a percentage of the vehicle-treated control. Data were analyzed using GraphPad Prism version 9.0 (RRID:SCR_002798).

### 2.10. Statistical Analysis

For experiments involving primary human monocytes derived from different donors, the donor was considered the biological unit of analysis. Each experimental condition was performed in technical triplicate, and at least three independent biological replicates (distinct donors) were included in all experiments. Data were analyzed and visualized using GraphPad Prism software (RRID: SCR_002798). Data distribution was assessed for normality using the Shapiro–Wilk test. Comparisons between two groups were performed using paired one-tailed Student’s *t*-tests, while comparisons among multiple groups were conducted using one-way analysis of variance (ANOVA) followed by Bonferroni post hoc test, as appropriate. Paired *t*-tests or repeated-measures (RM) ANOVA were applied to account for inter-individual biological variability. Results are expressed as mean ± standard deviation (SD), as indicated in the figure legends. Statistical significance was defined as *p* < 0.05.

## 3. Results

### 3.1. M. leprae Increases the Expression of Ectoenzyme CD39 in Human Monocytes

**Figure 1:**
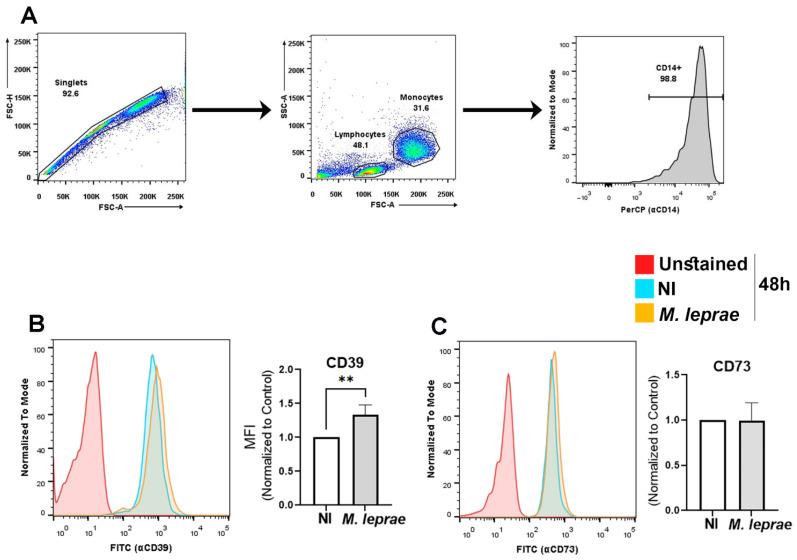
*M. leprae* increases CD39 expression in human monocytes. Enriched monocytes from PBMCs were infected with *M. leprae* (MOI 10:1) for 48 h, and the expression of CD39 and CD73 was assessed by flow cytometry. (**A**) Gating strategy: Singlets were selected based on FSC-A vs. FSC-H, followed by monocyte identification based on size (FSC-A) and granulosity (SSC-A). CD14^+^ monocytes were identified using anti-CD14 conjugated to PerCP Cy5.5, confirming a purity of approximately 98%. (**B**,**C**) Representative histograms showing CD39 (**B**) and CD73 (**C**) expression in unstained cells (red), non-infected (NI; cyan), and *M. leprae*-infected (orange) monocytes. Bar graphs represent quantification of median fluorescence intensity (MFI), normalized to NI controls. After normalization, data from the infection condition (*M. leprae*) are presented as mean ± SD from four independent donors. Each biological replicate was analyzed in technical duplicate. Statistical significance was assessed using a paired *t*-test. (** *p* < 0.01).

To investigate whether *M. leprae* infection modulates the expression of key enzymes regulating extracellular ATP and ADO levels, the expression of CD39 and CD73 was analyzed by flow cytometry. For this, human monocytes were isolated from PBMCs of healthy individuals and infected with *M. leprae* (MOI 10:1) for 48 h. The frequency of human monocytes expressing CD39 and CD73 was analyzed. Figure 1A shows the gating strategy used to identify CD14^+^ monocytes. The singlets were first selected (left graph), the gate of the monocyte population was based on size using Forward Scatter (FSC) versus granularity, Side Scatter (SSC) (middle graph), and finally, confirmed with the expression of CD14 (right graph), which revealed a purity of about 98%, which reflects the gated monocyte population for flow cytometry analysis, not total PBMC purity. Figure 1B,C show representative overlaid histograms (unstained, red; non-infected (NI), cyan; *M. leprae*, orange) of CD39 and CD73, respectively. The results show a rightward shift in CD39 expression with infection, whereas no shift in CD73 expression was observed between NI and infected cells. The bar graph (MFI, normalized to NI) indicates a significant increase in CD39 expression by approximately 33 ± 15%, when compared to NI (*p* = 0.0088; Figure 1B). In contrast, CD73 expression remained unchanged upon infection (Figure 1C). These findings suggest that *M. leprae* modulates extracellular ATP levels primarily by upregulating CD39, potentially as a pathogen-driven mechanism to limit ATP-mediated inflammatory responses in monocytes without altering AMP-to-ADO conversion.

### 3.2. CD73 Is a Potential Regulator of Lipid Droplet Formation in M. leprae-Infected Monocytes

Although CD73 expression did not change, we hypothesized that its enzymatic activity might still affect extracellular ADO levels. Since extracellular ADO can regulate lipid metabolism [24,42,60], we tested whether adding 5′-AMP, the CD73 substrate, would alter LD formation during *M. leprae* infection.

**Figure 2:**
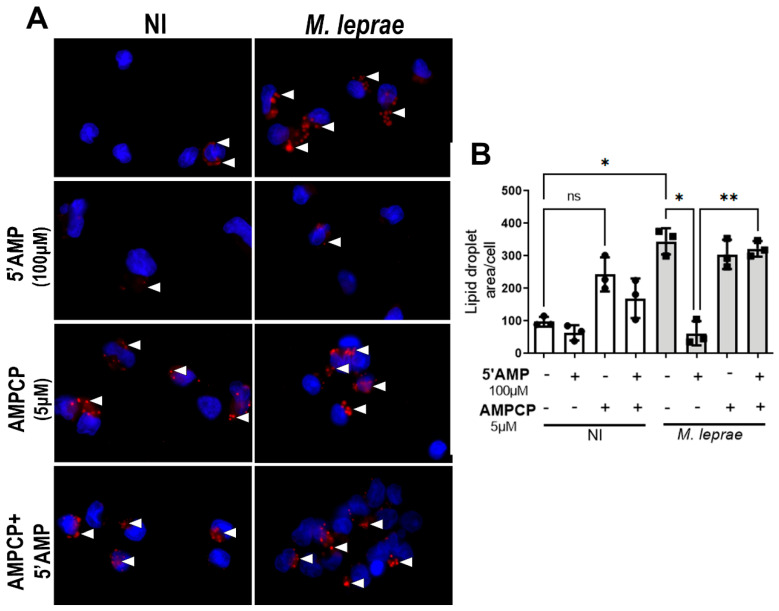
CD73 activity modulates lipid droplet formation in *M. leprae*-infected human monocytes. Enriched human monocytes from PBMCs were infected with *M. leprae* (MOI 10:1) for 48 h. (**A**) Representative fluorescence microscopy images of non-infected (NI) and *M. leprae*-infected human monocytes treated with 5′AMP (100 µM), AMPCP (5 µM), or their combination. LDs were stained with Oil Red O (red), and the LD area per cell was analyzed in a total of 200 cells per condition. Nuclei were stained with DAPI (blue). White arrowheads indicate LDs. Scale bar = 10 μm. (**B**) LD quantification was performed using ImageJ software. NI = not infected. Data are presented as mean ± SD from at least three independent experiments conducted in technical duplicate. Statistical significance was determined by paired one-way ANOVA followed by Bonferroni’s multiple comparisons test (* *p* < 0.05, ** *p* < 0.01, ns = not significant).

For this, human monocytes were pretreated with 5′-AMP (100 µM) and/or AMPCP (5 µM), a selective CD73 inhibitor, and then infected with *M. leprae*. LDs were stained with Oil Red O (ORO) and analyzed by fluorescence microscopy. The red-stained structures, indicated by white arrowheads, are visible in the representative images of monocytes shown in Figure 2A.

As expected, *M. leprae* infection markedly increased LD formation compared to NI, corroborating findings from previous studies [16,17,18,19,20,24]. The treatment with 5′AMP significantly reduced *M. leprae*-induced LD accumulation by approximately 81 ± 9% (*p* = 0.0298). This effect was abolished in infected cells in the presence of AMPCP (*p* = 0.0090) (Figure 2B). Although AMPCP treatment in the NI condition showed a trend toward increased LD area, this difference was not statistically significant (*p* = 0.1203). These results suggest that CD73 activity may play an important role in regulating lipid homeostasis during infection.

To ensure that the observed pharmacological effects were not due to cytotoxicity, we evaluated the impact of all compounds used in this study on cell viability. MTT assay results demonstrated that none of the treatments affected monocyte viability (Supplementary Figure S1).

### 3.3. Adenosine Downregulates Lipid Droplet Formation in Monocytes Infected by M. leprae

**Figure 3:**
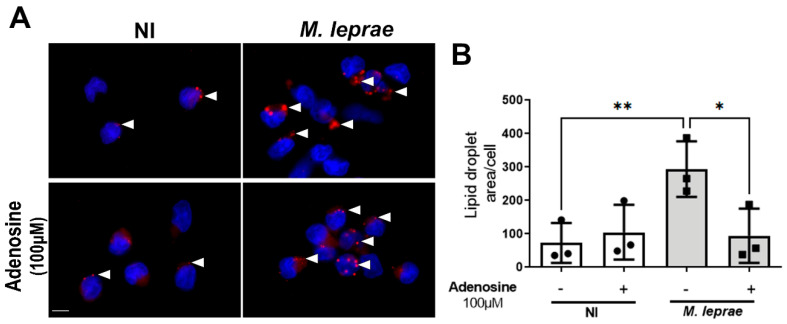
Treatment with ADO decreases lipid droplet levels in *M. leprae*-infected monocytes. Enriched human monocytes isolated from PBMCs were infected with *M. leprae* (MOI 10:1) for 48 h. (**A**) Representative fluorescence microscopy images of non-infected (NI) and *M. leprae*-infected human monocytes treated with ADO (100 µM). LDs were stained with Oil Red O (red), and the LD area per cell was analyzed in a total of 200 cells per condition. Nuclei were stained with DAPI (blue). White arrowheads indicate LDs. Scale bar = 10 μm. (**B**) LD quantification was performed using ImageJ software. NI = not infected. Data are presented as mean ± SD from at least three independent experiments conducted in technical duplicate. Statistical significance was determined by paired one-way ANOVA followed by Bonferroni’s multiple comparisons test (* *p* < 0.05, ** *p* < 0.01).

Since CD73 hydrolyzes 5′-AMP to generate ADO, we next evaluated whether ADO *per se* could produce a similar effect to that observed following 5′-AMP addition. To confirm this hypothesis, *M. leprae*-infected monocytes were pretreated with ADO (100 µM) followed by infection with *M. leprae* and staining of LDs with ORO. Treatment with ADO significantly reduced LD accumulation, as evidenced by a decreased LD area (Figure 3A). Quantification revealed a reduction by approximately 68 ± 44% LD area in *M. leprae*-infected monocytes treated with ADO compared to infected monocytes without treatment (*p* = 0.0124; Figure 3B), supporting our hypothesis that extracellular ADO regulates lipid metabolism during *M. leprae* infection.

### 3.4. M. leprae Infection Differentially Modulates the Expression of Adenosine Receptors in Human Monocytes

**Figure 4:**
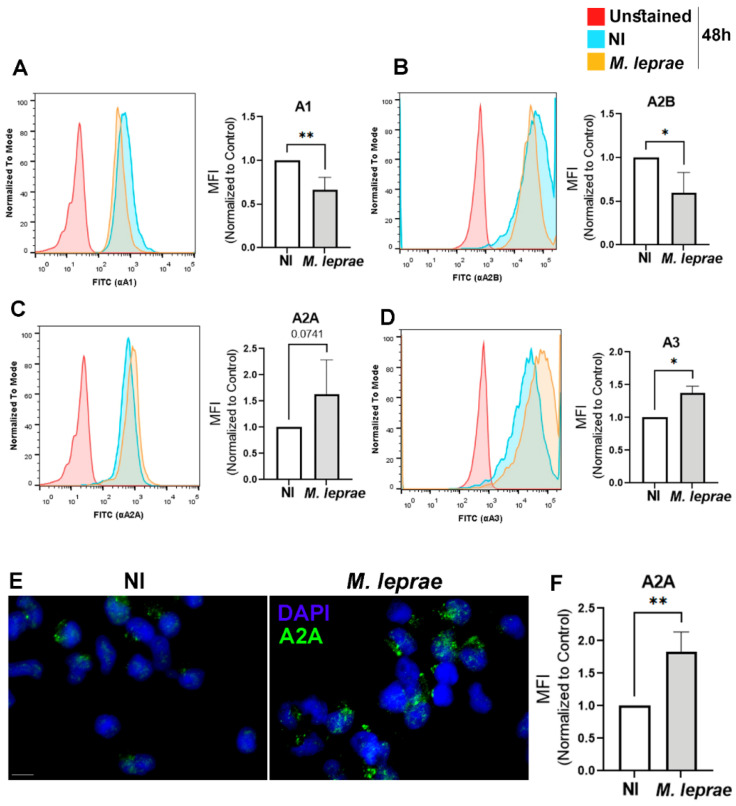
*M. leprae* infection selectively modulates ADO receptor expression in monocytes. Enriched human monocytes isolated from PBMCs were infected with *M. leprae* (MOI 10:1) for 48 h. The expression of A_1_, A_2B_, A_3,_ and A_2A_ was assessed by flow cytometry and fluorescence microscopy. (**A**–**D**) Representative histograms showing (**A**) A_1_R, (**B**) A_2B_R, (**C**) A_2A_R, and (**D**) A_3_R expression in unstained cells (red), non-infected (NI; blue), and *M. leprae*-infected (orange) monocytes. (**E**) Representative fluorescence microscopy images of non-infected (NI) and *M. leprae*-infected human monocytes. A total of 200 cells per condition were stained with DAPI (blue) and an anti-A_2A_ antibody (green). (**F**) Bar graphs represent quantification of median fluorescence intensity (MFI), normalized to NI controls. Data are presented as mean ± SD from four independent donors, with each measurement conducted in duplicate. Scale bar = 10 μm. Statistical significance was determined using a paired *t*-test (* *p* < 0.05, ** *p* < 0.01).

Extracellular ADO modulates a wide range of cellular processes by activating P1 receptors, specifically A_1_, A_2A_, A_2B_, and A_3_ [26,27]. The next goal was to investigate the expression profile of P1 receptors in *M. leprae*-infected monocytes. Flow cytometry analysis revealed that infected monocytes exhibited a 34 ± 10% reduction in A_1_R expression (*p* = 0.009; Figure 4A) and a 41 ± 17% reduction in A_2B_R expression (*p* = 0.0475; Figure 4B) compared with NI cells. However, A_3_R expression was upregulated by 37 ± 8% in infected monocytes (*p* = 0.0112; Figure 4D). For A_2A_R, flow cytometry showed an increase in infected cell expression (Figure 4C), which was confirmed by immunofluorescence, revealing an 83 ± 30% increase compared with the NI condition (*p* = 0.0057; Figure 4E,F).

### 3.5. A_2A_ Receptor Negatively Regulates Lipid Droplet Formation in Human Monocytes Infected by M. leprae

**Figure 5:**
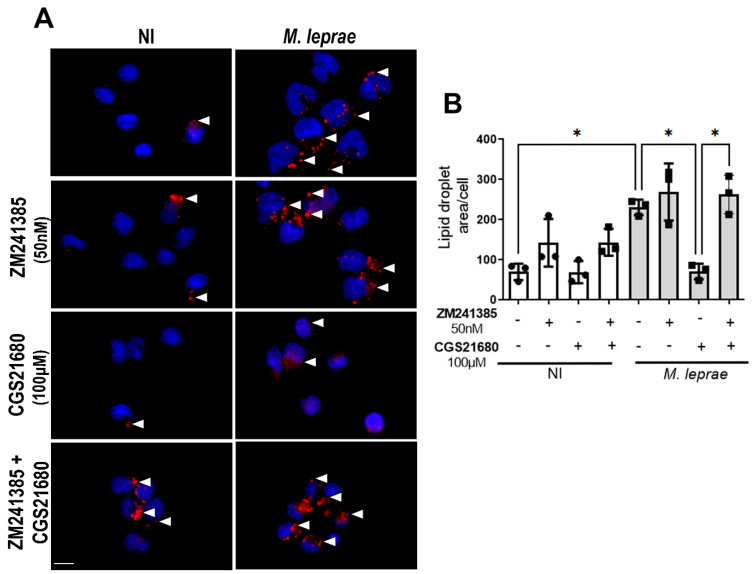
Activation of the A_2A_R decreases lipid droplet formation in *M. leprae*-infected human monocytes. Enriched human monocytes isolated from PBMCs were pretreated and infected with *M. leprae*. (**A**) Representative fluorescence microscopy images of non-infected (NI) and *M. leprae*-infected human monocytes treated with CGS21680 (100 µM) and or ZM241385 (50 nM). LDs were stained with Oil Red O (red), and the LD area per cell was analyzed in a total of 200 cells per condition. Nuclei were stained with DAPI (blue). White arrowheads indicate LDs. Scale bar = 10 μm. (**B**) LD quantification was performed using ImageJ software. NI = not infected. Data were presented as mean ± SD from at least three independent experiments conducted in technical duplicate. Statistical significance was determined by paired one-way ANOVA followed by Bonferroni’s multiple comparisons test (* *p* < 0.05).

Previous studies have reported that activation of the A_2A_R is associated with reduced intracellular cholesterol accumulation [43,44,45]. Our group previously demonstrated that the A_2A_R agonist CGS21680 (100 µM) reduced the LD formation in Schwann cells infected with *M. leprae* [24]. To investigate whether this phenomenon also occurs in monocytes, cells were pretreated with a specific A_2A_R agonist (CGS21680) and infected as described before. A dose–response analysis was performed with CGS21680 at 50 nM, 1 µM, 10 µM, and 100 µM, all of which yielded a comparable reduction in lipid droplet (LD) formation (Supplementary Figure S2). Therefore, to maintain consistency with our previous studies, we started the analysis by evaluating the effect of 100 µM CGS21680. The results showed that the treatment of monocytes with the A_2A_R agonist CGS21680 (100 µM) reduced LD accumulation by 68 ± 12% in infected cells (*p* = 0.0297; Figure 5A,B). Moreover, inhibition of A_2A_R with the selective antagonist ZM241385 (50 nM) reversed this effect (*p* = 0.0319; Figure 5B).

### 3.6. CGS21680 Reduces Lipid Droplet Formation in an A_2B_R–Independent Manner

**Figure 6:**
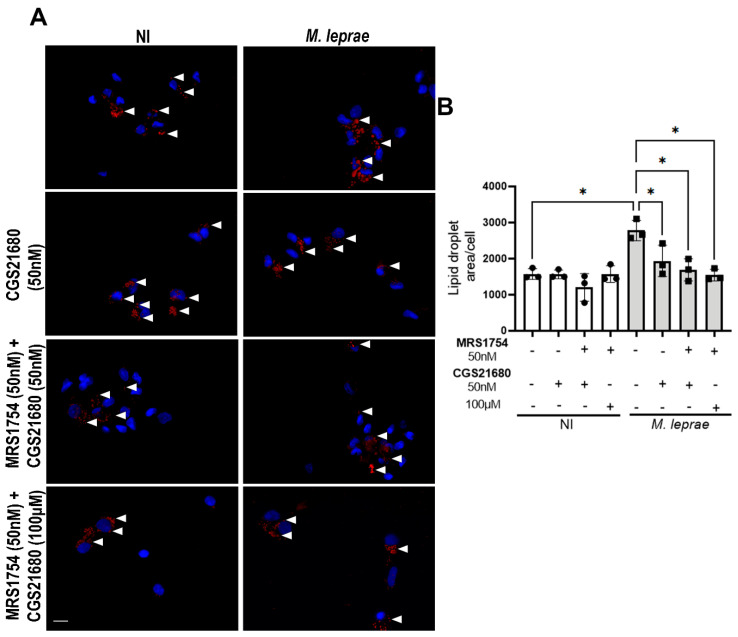
CGS21680 reduces lipid droplets in *M. leprae-infected* monocytes independently of A_2B_R. Enriched human monocytes isolated from PBMCs were infected with *M. leprae* (MOI 10:1) for 48 h. (**A**) Representative fluorescence microscopy images of non-infected (NI) and *M. leprae*-infected human monocytes treated with CGS21680 (50 nM, 100 µM), MRS1754 (50 nM), or their combination. LDs were stained with Oil Red O (red), and the LD area per cell was analyzed in a total of 200 cells per condition. Nuclei were stained with DAPI (blue). White arrowheads indicate LDs. Scale bar = 10 μm. (**B**) LD quantification was performed using ImageJ software. NI = not infected. Data are presented as mean ± SD from at least three independent experiments conducted in technical duplicate. Statistical significance was determined by paired one-way ANOVA followed by Bonferroni’s multiple comparisons test (* *p* < 0.05).

To exclude potential A_2B_R-mediated effects associated with high concentrations of CGS21680, LD biogenesis was evaluated during infection using a lower concentration of CGS21680 (50 nM) in the presence of the selective A_2B_R antagonist MRS1754. Consistent with the effects observed at 100 µM, treatment with 50 nM CGS21680 significantly reduced LD levels in infected cells by 28 ± 9% (*p* = 0.0470) (Figure 6). Moreover, in agreement with previous reports [24], MRS1754 did not interfere with the effects of CGS21680 at either 50 nM (*p* = 0.0351) or 100 µM (*p* = 0.0215) (Figure 6B). Together, these results indicate that the reduction in LD levels observed in *M. leprae*-infected monocytes is mediated by A_2A_R activation.

### 3.7. Activation of the A_2A_R Reverses the Loss of Mitochondrial Membrane Potential in M. leprae-Infected Monocytes

**Figure 7:**
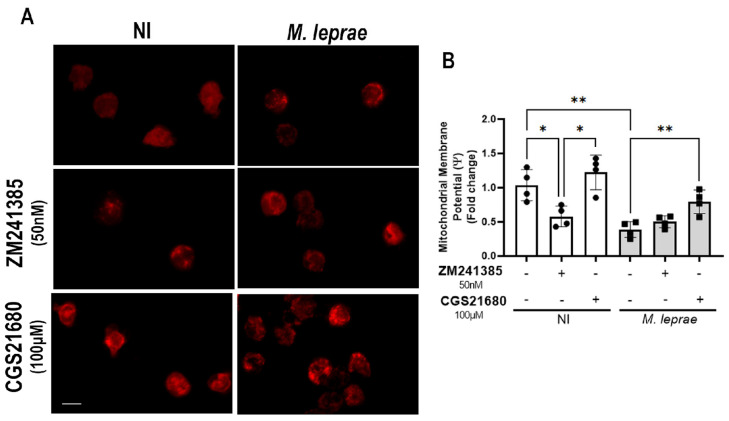
Treatment with CGS21680 increases mitochondrial membrane potential in *M. leprae*-infected monocytes. Human monocytes enriched from PBMCs were pretreated and infected with *M. leprae* (MOI 10:1 for 48 h) (**A**) Representative fluorescence microscopy images of non-infected (NI) and *M. leprae*-infected human monocytes treated with CGS21680 (100 µM) or ZM241385 (50 nM). Δψm was assessed using a mitochondrial fluorescent probe (TMRM) using a total of 200 cells per condition (red). Scale bar: 10 µm. (**B**) Quantification of Δψm (fold change) in different conditions. Data are presented as mean ± SD from four independent experiments conducted in duplicate. Statistical differences were evaluated using one-way ANOVA followed by Bonferroni’s multiple comparisons test (* *p* < 0.05; ** *p* < 0.01).

Since activation of the A_2A_R exerts a mitoprotective effect by upregulating Δψm [43,44,45], and *M. leprae* infection downregulates Δψm in Schwann cells and monocytes [13,57], we wondered whether A_2A_R activation could reverse the reduction in Δψm in monocytes infected with *M. leprae*. Thus, we treated monocytes with CGS21680 (100 µM) or ZM241385 (50 nM), infected or not with *M. leprae*, and analyzed mitochondrial fluorescence using TMRM, a Δψm-sensitive probe, by fluorescence microscopy. The result shows that in the NI condition, the inhibition of A_2A_R with ZM241385 reduced the Δψm by 44 ± 15% compared with untreated NI cells (*p* = 0.0195). In contrast, treatment with CGS21680 increased the Δψm by approximately 110% ± 38% when compared with ZM241385-treated cells (*p* = 0.0187) (Figure 7A). In monocytes infected with *M. leprae*, Δψm decreased by 70 ± 6% (*p* = 0.0065) compared with the NI condition, corroborating our previously published findings [13]. Additionally, the addition of CGS21680 restored Δψm in infected cells, promoting an approximately 2-fold increase compared with untreated infected monocytes (*p* = 0.0150) (Figure 7B). Together, these data indicate that A_2A_R sustains mitochondrial function at baseline in NI cells and that pharmacologic A_2A_R activation reverses the *M. leprae*-induced mitochondrial depolarization in human monocytes.

### 3.8. Activation of the A_2A_ Receptor Decreases the Intracellular Viability of M. leprae

**Figure 8:**
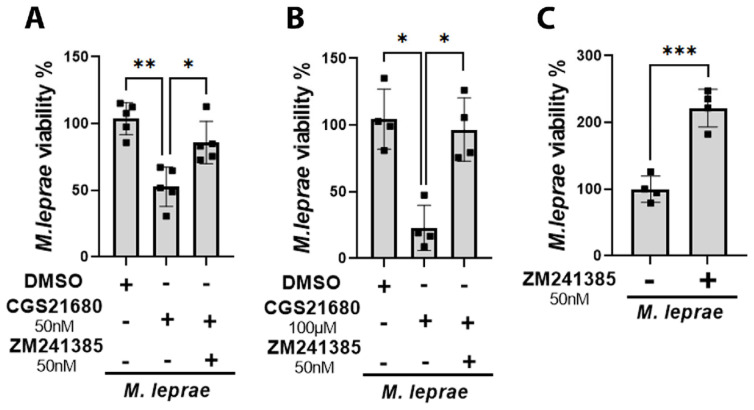
A_2A_R activation reduces *M. leprae* viability in human monocytes. Enriched human monocytes isolated from PBMCs were infected with *M. leprae* (MOI 10:1) for 48 h, and pretreated or not with the selective A_2A_R agonist CGS21680 (50 nM or 100 µM) and/or the antagonist ZM241385 (50 nM). *M. leprae* viability was assessed by RT-qPCR using the ratio of 16S cDNA to 16S DNA represented by percentage relative to the control. (**A**,**B**) Pretreatment with CGS21680 significantly reduced *M. leprae* viability, and this effect was reversed by co-treatment with ZM241385. (**C**) Treatment with ZM241385 alone increased the bacterial survival compared to untreated infected cells. Data are presented as mean ± SD relative to the control, from at least four independent experiments in technical duplicate. Statistical significance was determined using one-way ANOVA followed by Bonferroni’s multiple comparisons test or using a paired *t*-test (* *p* < 0.05; ** *p* < 0.01; *** *p* < 0.001).

We showed that A_2A_R activation reverses two important metabolic alterations in *M. leprae*-infected cells that contribute to bacillary persistence and the establishment of infection: accumulation of LD and a reduction in Δψm. To investigate whether these effects of A_2A_R activation could affect intracellular *M. leprae* viability, we treated *M. leprae*-infected cells with 50 nM and 100 µM of CGS21680 and assessed bacterial viability by RT-qPCR. The results revealed that, at both doses, CGS21680 reduced M. leprae viability by about 48 ± 14% at 50 nM (*p* = 0.0039) and 77 ± 16% at 100 μM (*p* = 0.0056) compared to the vehicle (DMSO). However, this effect was reverted by the A_2A_R antagonist ZM241385 (50 nM), resulting in a 2.7-fold increase in *M. leprae* survival (*p* = 0.0031) (Figure 8A–C).

### 3.9. Adenosinergic Compounds Reduce Lipid Droplet Accumulation Independently of M. leprae Viability

**Figure 9:**
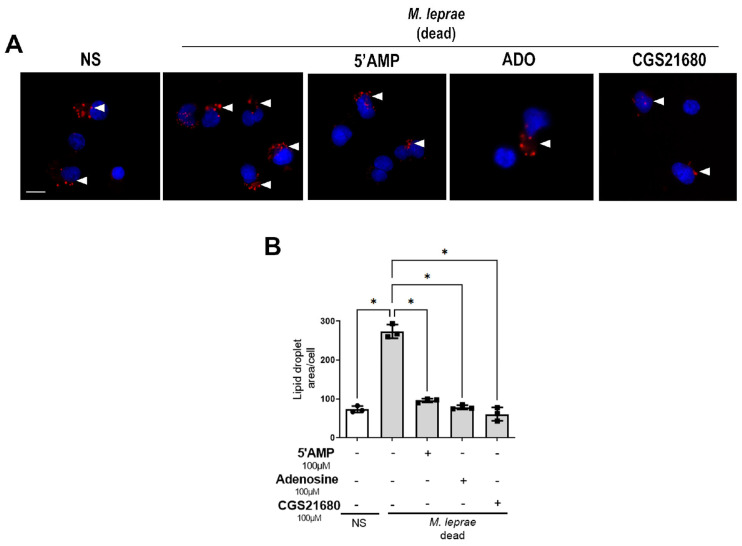
Lipid droplet reduction by adenosinergic compounds is independent of *M. leprae* viability. Enriched human monocytes isolated from PBMCs were stimulated with dead *M. leprae* (MOI 10:1) for 48 h, and (**A**) representative fluorescence microscopy images of non-stimulated (NS) and *M. leprae*-stimulated human monocytes treated with 5′AMP, ADO or CGS21680 (100 µM). LDs were stained with Oil Red O (red), and the LD area per cell was analyzed in a total of 200 cells per condition. Nuclei were stained with DAPI (blue). White arrowheads indicate LDs. Scale bar = 10 μm. (**B**) LD quantification was performed using ImageJ software. Data are presented as mean ± SD from three independent experiments conducted in duplicate. Statistical significance was determined by paired one-way ANOVA followed by Bonferroni’s multiple comparisons test (* *p* < 0.05).

Considering that treatment with CGS21680 decreases *M. leprae* viability (Figure 8A,B), we hypothesized that the reduced formation of LDs observed in the presence of 5′-AMP, ADO, and CGS21680 during *M. leprae* infection may be a consequence of decreased bacterial viability rather than a direct inhibition of LD biogenesis. However, our group has previously shown that macrophages stimulated with dead *M. leprae* are still able to induce lipid droplet formation [19]. To determine whether human monocytes exhibit a similar phenotype, we stimulated monocytes with dead *M. leprae* and treated them with 5′-AMP, ADO, or CGS21680. The stimulation with dead *M. leprae* increased LD area by 3.7-fold when compared to non-stimulated cells (NS) (*p* = 0.0175) and treatment with 5′AMP, ADO, and CGS21680 reduced the LD area by 59 ± 5% (*p* = 0.0246), 64 ± 5% (*p* = 0.0259), and 79 ± 17% (*p* = 0.0343) respectively, suggesting that the downregulation of LDs by adenosinergic compounds occurs independently of bacillary viability (Figure 9A,B).

### 3.10. M. leprae-Infected Monocytes Downregulate the Expression of ENT1, Exhibit Higher ADA Expression and Extracellular Inosine and Hypoxanthine Levels

**Figure 10:**
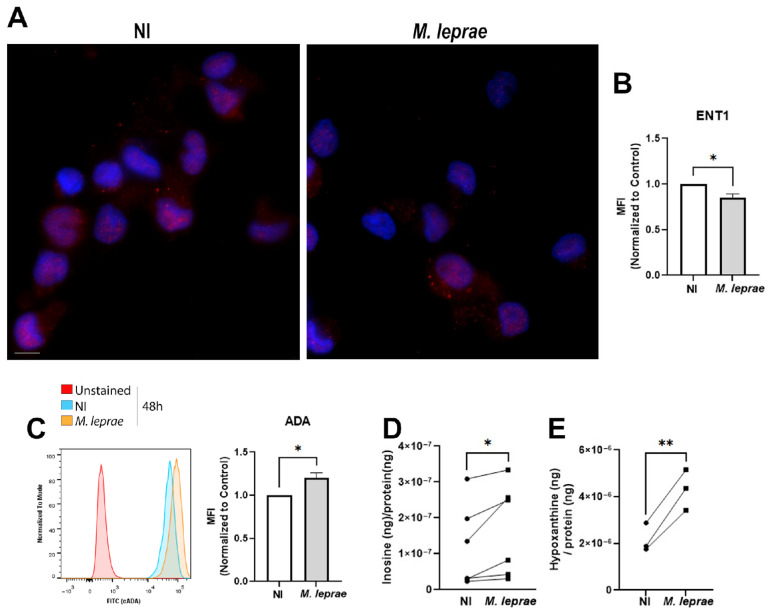
*M. leprae* infection decreases ENT1 and upregulates ADA expression, leading to higher extracellular inosine and hypoxanthine levels. Human monocytes enriched from PBMCs were infected with *M. leprae* (MOI 10:1) for 48 h, and ADA expression was assessed by flow cytometry, and nucleoside levels were measured by LC-MS/MS. (**A**) Representative fluorescence microscopy images of non-infected (NI) and *M. leprae*-infected human monocytes. Cells were stained with DAPI (blue) and an anti-ENT1 antibody (red) and analyzed from a total of 200 cells per condition. (**B**) The bar graph represents quantification of median fluorescence intensity (MFI) normalized to NI controls, demonstrating a significant decrease in ENT1 expression upon infection (* *p* < 0.05). Scale bar = 10 μm. (**C**) Representative histogram shows ADA expression in unstained cells (red), non-infected (NI, blue), and *M. leprae*-infected cells (orange). Quantification of the mean fluorescence intensity (MFI) normalized to control demonstrates a significant increase in ADA expression upon infection (* *p* < 0.05). (**D**,**E**) Quantification of extracellular levels of inosine and hypoxanthine in infected and non-infected cells. LC-MS/MS data were normalized to protein levels and are expressed as ng/ng. Data represents SD from at least three independent experiments. Statistical significance was determined using a paired *t*-test (* *p* < 0.05, ** *p* < 0.01).

These results suggest that A_2A_R activation may influence metabolic responses in host cells infected with *M. leprae*. However, infection was also associated with increased A_2A_R expression, suggesting that A_2A_R signaling may be differentially regulated during infection. Therefore, we examined the effect of *M. leprae* infection on the expression of the equilibrative transporter ENT1 and the ectoenzyme ADA, which mediate the reuptake of ADO/INO and the deamination of ADO to INO, respectively. In addition, we investigated the influence of the infection on the extracellular ADO, INO, and HPX levels. Human monocytes were infected with *M. leprae* as described earlier, and immunofluorescence analysis showed a downregulated expression of ENT1 by 15 ± 3% (*p* = 0.0101; Figure 10A,B), and flow cytometry analysis showed that *M. leprae* infection increased ADA expression by 21 ± 5% compared with NI cells (*p* = 0.0123; Figure 10C). To validate this result, we performed LC-MS/MS analysis of supernatants from these cells to determine extracellular levels of purinergic mediators. Although we had not detected extracellular ADO in either condition, we observed increased INO and HPX in the supernatant of infected cells compared with NI (Figure 10D,E), suggesting high activity of ADA and purine nucleoside phosphorylase (ecto-PNP). Interestingly, our findings highlight that *M. leprae* infection not only alters receptor signaling A_2A_R but also, for the first time, shows that it rewires extracellular purine metabolism, and reveals new mechanisms that may be contributing to the chronic persistence and tissue pathology of leprosy.

## 4. Discussion

This work highlights the role of A_2A_R in the interaction between *M. leprae* and monocytes. Despite increased expression in A_2A_R and A_3_R, we focus on A_2A_R due to its anti-lipogenic and mitoprotective effects [24,42,43,44,45]. Our findings suggest that *M. leprae* bypasses these effects by increasing ADA expression, which downregulates extracellular ADO levels and thereby potentially suppresses A_2A_R activation in infected monocytes. In addition, increased expression of the ADA enzyme, as well as extracellular levels of INO and HPX in infected cells, support the idea that extracellular ADO levels decrease during infection and that INO and HPX can play a relevant role in the *M. leprae*-host cell interaction.

Monocytes are key immune cells involved in the clearance of invading pathogens, and the purinergic system has been implicated in host–pathogen interactions [8,30]. Recent research highlights this system as a promising target for metabolic interventions in the treatment of chronic inflammatory and infectious diseases [30,61]. The study with naïve monocytes in vitro contributes to unraveling the early immunomodulatory mechanisms of circulating monocytes, helping to understand the immune tolerance observed in leprosy.

In microenvironments of infected cells, elevated extracellular ATP levels are commonly observed [62,63]. Extracellular ATP is an essential molecule for paracrine signaling, modulating innate immunity, and enhancing macrophage phagocytosis through P2X7- and P2X4-dependent pathways [64]. However, extracellular ATP also activates inflammasomes and triggers intense pro-inflammatory responses, contributing to microbicidal activity [65,66]. For example, CD39-deficient macrophages exhibit a pro-inflammatory phenotype and fail to adopt an immunosuppressive state [67]. Thus, *M. leprae* infection may increase CD39 expression in host cells, thereby regulating extracellular ATP levels to favor its uptake. Although CD39 levels increased in *M. leprae*-infected monocytes, the increase was moderate (~33%) and varied between donors. Even small changes, however, may affect function because CD39 is key in breaking down ATP. Thus, modest increases may shift the balance between ATP and ADO, controlling both inflammation and immune suppression. Greater ATP breakdown could lower inflammatory signals and enhance ADO’s role in immune tolerance. These metabolic shifts are also linked to increased lipid droplet formation and pathogen persistence. The observed differences likely reflect natural variation in cells from different people, which is common in ex vivo infection studies. Despite this variability, the effect was still statistically significant, underscoring its importance. However, future studies assessing CD39 enzymatic activity will be important to determine how this enzyme regulates the extracellular ATP–ADO balance and how this modulation may influence immune responses in infected monocytes.

In the inflammatory microenvironment, the tight regulation of the extracellular ATP/ADO ratio becomes a key mechanism by which pathogens subvert host immune responses, mainly through the metabolic reprogramming of innate and adaptive immune cells [30]. We have not observed any change in CD73 expression in *M. leprae*-infected monocytes; however, treatment with its substrate, 5′AMP, induces an anti-lipogenic effect in infected cells, which was abolished in the presence of the specific enzymatic inhibitor, as observed by Cui and colleagues [35], reinforcing CD73′s functional activity in our model. Nonetheless, further studies are needed to evaluate the real impact of *M. leprae* infection on the activity of CD73 and its role in leprosy pathogenesis.

5′-AMP has been shown to activate both A_1_R and A_2A_R [68,69], receptors involved in lipid metabolism [42]. Overall, these results indicate that *M. leprae* infection modulates CD73 activity to regulate extracellular 5′AMP/ADO levels, thereby maintaining the pro-lipid intracellular profile in the host cell. In this study, we observed that the addition of ADO reduced LD formation induced by *M. leprae* infection, as previously reported in infected Schwann cells [24]. Furthermore, we confirmed the effect of A_2A_R pharmacologically using CGS21680 (an agonist) and ZM241385 (an antagonist). We selected the concentration of CGS21680 based on previous studies. Yasuda et al. (2003) and Lebon et al. (2015) employed 100 µM in hepatocytes and in receptor structural analyses, respectively, demonstrating high selectivity for A_2A_R over A_2B_R [70,71]. Furthermore, our group also used 100 µM CGS21680 in *M. leprae*-infected Schwann cells, demonstrating its effect on lipid metabolism [24]. Thus, we maintained this concentration for comparability with past data. Since A_2A_R activation occurs in the nanomolar to micromolar range [24,70,71], we also tested 50 nM CGS21680 and included an A_2B_R antagonist (MRS1735) to assess specificity. The A2BR antagonist did not affect the anti-lipogenic effect induced by either 50 nM or 100 µM CGS21680, strengthening evidence for CGS21680′s specificity for A_2A_R.

The use of 100µM of CGS21680 was a pharmacological strategy to achieve maximal stimulation conditions designed to (i) ensure robust A_2A_ pathway engagement in a milieu where *M. leprae* increases ADA and alters nucleoside handling, reducing the effective extracellular ADO concentration and (ii) stress-test specificity alongside selective antagonists.

Although the precise molecular mechanisms were not examined in the present study, activation of A_2A_R is known to elevate intracellular cAMP levels and engage the PKA–CREB signaling axis, which has been associated with coordinated anti-lipogenic and metabolic regulatory programs [72]. For example, PKA accelerates triglyceride hydrolysis by phosphorylating the hormone-sensitive lipase and perilipin-1 [73]. When perilipin-1 is phosphorylated, the surface of the LD changes, making it easier for lipolytic enzymes to attach. At the same time, PKA phosphorylation activates HSL, which moves from the cytosol to the lipid droplet, where it breaks down stored triglycerides into diglycerides and free fatty acids. These actions work together to increase lipolysis and reduce the amount of stored fat in the cell [74,75,76,77]. In addition, CREB activation can also influence cellular energy metabolism by regulating PGC-1α (peroxisome proliferator-activated receptor gamma coactivator-1 alpha), a master regulator of mitochondrial biogenesis and oxidative metabolism [78,79]. Upon phosphorylation by PKA, CREB binds to cAMP-responsive elements in the promoter region of the *PPARGC1A* gene, increasing the expression of PGC-1α, which in turn, acts as a transcriptional coactivator for nuclear receptors and transcription factors such as PPARs and NRF1/NRF2, which regulate genes involved in fatty acid β-oxidation, mitochondrial respiration, and oxidative phosphorylation [80]. Through this pathway, CREB-dependent induction of PGC-1α enhances mitochondrial function and promotes fatty acid utilization as an energy source, thereby contributing to reduced intracellular lipid accumulation. This pathway may help explain how A_2A_R activation restores mitochondrial membrane potential that is reduced during *M. leprae* infection.

In macrophages, the cAMP–PKA–CREB axis also increases ABCA1, enhancing cholesterol efflux to apoA-I [81,82,83]. Further, CREB induces ATF3, which binds the *PPAR*γ promoter and suppresses PPARγ transcription and target-gene expression [84,85,86]. Beyond transcription, cAMP/PKA can activate the MAPK/ERK pathway [87], which phosphorylates PPARγ, reducing its transactivation [88,89]. In summary, the cAMP–PKA–CREB pathway activated by A_2A_R may reduce LD accumulation by shifting cellular metabolism toward oxidation, providing a mechanistic basis for the anti-lipogenic phenotype observed during *M. leprae* infection. In addition, we observed in *M. leprae*-infected Schwann cells an anti-lipogenic effect following A_2A_R activation that involved the downregulation of key genes controlling lipid metabolism, such as *PPARγ*, *CD36*, and *PLIN3*, while simultaneously promoting the upregulation of *ABCA1* and *ABCG1*, which encode critical proteins involved in reverse cholesterol transport, as described in the literature [24]. Another relevant aspect of A_2A_R activation is its potential impact on the formation of lipid mediators induced during *M. leprae* infection. A_2A_R signaling is an important regulator in eicosanoid biology. When activated, A_2A_R engagement decreases pro-inflammatory eicosanoid production, including COX-2-derived prostaglandins and 5-lipoxygenase products [90,91]. Different clinical forms of leprosy exhibit distinct eicosanoid profiles, including prostaglandin E2, prostaglandin D2, and lipoxin A4 [92,93,94]. This supports the role of lipid mediators in the disease’s immunopathology. In addition, LDs induced by *M. leprae* are platforms for eicosanoid synthesis [19]. These molecules can act in various ways; for example, hemozoin produces 15(S)-HETE, a PPAR-γ ligand that inhibits dendritic cell differentiation and maturation [95]. In addition to its effects on cAMP-dependent signaling pathways, activation of the A_2A_R may additionally influence the metabolism of arachidonic acid-derived lipid mediators. Considering that LDs are key intracellular platforms for eicosanoid synthesis, modulation of these metabolic pathways by A_2A_R signaling may indirectly alter LD activity and the production of inflammatory lipid mediators during infection. In the context of *Mycobacterium leprae* infection, where LD accumulation and host lipid metabolism are central features of the host–pathogen interaction, A_2A_R-dependent regulation of these pathways may contribute to remodeling cellular lipid metabolism and inflammatory responses.

A_2A_R is also known to regulate mitochondrial function through cAMP/PKA signaling, which can either stabilize the mitochondrial function and ROS production [96,97]. Previous studies have demonstrated that A_2A_R and A_2B_R activation inhibit mitoROS production in chondrocytes and neutrophils, respectively [96,98]. In macrophages, A_2A_R activation promotes a metabolic shift toward oxidative phosphorylation and an anti-inflammatory phenotype, partially mediated by modulation of mitochondrial activity [97]. Additionally, increased electron transport-driven mitoROS production has been associated with enhanced pathogen clearance [99,100]. The mitochondrial function is essential for clearing pathogens and plays a central role in balancing lipid storage and fatty acid oxidation [101]. Furthermore, activation of A_2A_R may modulate mitochondrial functions, potentially leading to reduced intracellular viability of *M. leprae*. Together, these transcriptional and post-translational mechanisms may converge to suppress lipogenic pathways, enhance lipid catabolism, and improve mitochondrial fitness, ultimately counteracting the immunometabolic reprogramming induced by *M. leprae* infection. Ongoing studies are currently being conducted to further delineate the molecular pathways activated downstream of A_2A_R signaling in infected cells, with particular focus on cAMP-dependent networks, lipid metabolic regulators, and mitochondrial homeostasis.

The ATP/ADO ratio acts as an immunological switch, mediating transitions between pro- and anti-inflammatory responses [33]. In our model, *M. leprae* infection increased CD39 and ADA expression, which can be linked to the regulation of ATP/ADO levels. We also found that *M. leprae* infection decreases ENT1 expression in human monocytes, a nucleoside transporter responsible for the reuptake of ADO and INO [50]. Previous studies indicate that extracellular ADO levels can modulate ENT1 expression [102]. Thus, in infected monocytes, it is plausible that increased ADA activity reduces extracellular ADO, contributing to ENT1 downregulation. Nevertheless, additional studies are required to elucidate the specific contribution of extracellular purinergic components to the host cell microenvironment.

Our data demonstrated that *M. leprae* infection led to a significant increase in extracellular levels of INO and HPX, two purine metabolites implicated in lipid metabolism. Previous studies have shown that INO upregulates 3-hydroxy-3-methylglutaryl-CoA reductase (HMGCR), a key enzyme in cholesterol biosynthesis [103], whereas HPX downregulates APOE and ABCA1, both of which are crucial for cholesterol efflux and transport [104]. These findings suggest that *M. leprae* infection modulates the catabolic pathway of purine metabolites, thereby altering the host extracellular microenvironment to favor cholesterol accumulation. Such metabolic reprogramming likely supports LD formation and creates a lipid-enriched niche that benefits bacterial persistence.

Our findings demonstrate that activation of the A_2A_R by CGS21680 reduces LD accumulation in *M. leprae*-infected monocytes and restores Δψm. This suggests that A_2A_R signaling may shift the metabolic profile toward increased lipid catabolism and mitochondrial activation. Given that LDs provide a lipid-rich niche that supports *M. leprae* intracellular survival, A_2A_R-induced LD reduction and mitochondrial reactivation likely should contribute to limiting bacterial viability. To confirm this hypothesis, intracellular viability assays were performed, and we observed that treatment with CGS21680 significantly reduced bacterial viability, and the addition of ZM241385 reversed this effect. Interestingly, when we added only ZM241385, we detected increased *M. leprae* survival. These results suggest that, in a microenvironment enriched in extracellular ADO may be engaged to limit bacillary survival; conversely, when A_2A_R is pharmacologically blocked, extracellular ADO may become available to activate other adenosine receptor subtypes, which might promote *M. leprae* persistence. We cannot rule out the possibility that compensatory activation by other adenosine receptors also contributes to the observed effects. While our data support a central role for A_2A_R signaling in regulating the immunometabolic response of *M. leprae*-infected monocytes, the involvement of additional ADO receptor subtypes cannot be excluded. Monocytes and macrophages express all four adenosine receptors, which may be differentially engaged depending on extracellular ADO levels and the inflammatory environment [105]. Therefore, ongoing studies are exploring the role of alternative ADO receptors in host–pathogen interactions to better understand the adenosinergic axis’s involvement in leprosy pathogenesis.

## 5. Conclusions

Our findings show that *M. leprae* infection triggers coordinated immunometabolic changes in human monocytes. It remodels the extracellular purinergic environment, as evidenced by increased ATP hydrolysis, higher ADA activity, and the buildup of metabolites such as inosine and hypoxanthine, which affect lipid and cholesterol metabolism. Infected monocytes also display more LDs and mitochondrial dysfunction. These changes create an intracellular microenvironment that supports bacillary survival. In this context, A_2A_R acts as a metabolic checkpoint. It increases lipid breakdown and restores mitochondrial function, shifting metabolism to limit pathogen survival. Our data thus highlight purinergic signaling as an upstream regulator connecting extracellular nucleotide processing to intracellular lipid storage, mitochondrial function, and host defense, and targeting the purinergic signaling axis as a potential new therapeutic opportunity to enhance immune control and limit bacterial persistence in leprosy.

## Data Availability

The original contributions presented in this study are included in the article/Supplementary Materials. Further inquiries can be directed to the corresponding author.

## Supplementary Materials

The following supporting information can be downloaded at: https://www.mdpi.com/article/10.3390/metabo16050304/s1. Supplementary Figure S1. Treatment with adenosinergic pathway components does not affect monocyte viability. Supplementary Figure S2. Dose–response analysis of CGS21680 on lipid droplet accumulation in *M. leprae*-infected human monocytes.

## Abbreviations

The following abbreviations are used in this manuscript:

ADO	Adenosine
5′AMP	Adenosine monophosphate
INO	Inosine
A_1_	Adenosine A1 receptor
A_2A_R	Adenosine A2A receptor
A_3_	Adenosine A3 receptor
A_2B_R	Adenosine A2B receptor
ADA	Adenosine deaminase
AMPCP	α,β-methylene adenosine 5′-diphosphate
CCCP	Carbonyl cyanide m-chlorophenyl hydrazone
BSA	Bovine serum albumin
ANOVA	Analysis of variance
CEP	Research Ethics Committee
CEUA	Institutional Animal Care and Use Committee
cDNA	Complementary DNA
PBMCs	Peripheral blood mononuclear cells
DAPI	4′,6-diamidino-2-phenylindole
DMSO	Dimethyl sulfoxide
ENT-1	Equilibrative nucleoside transporter 1
HPX	Hypoxanthine
IF	Immunofluorescence
PFA	Paraformaldehyde
LC-MS/MS	Liquid chromatography–tandem mass spectrometry
LDs	Lipid droplets
FBS	Fetal bovine serum
MOI	Multiplicity of infection
ESI	Electrospray ionization
FIOCRUZ	Oswaldo Cruz Foundation
MFI	Median fluorescence intensity
MTT	3-(4,5-dimethylthiazol-2-yl)-2,5-diphenyltetrazolium bromide
PBS	Phosphate-buffered saline
RM ANOVA	Repeated-measures analysis of variance
RT-qPCR	Reverse transcription quantitative polymerase chain reaction
SD	Standard deviation
SDS	Sodium dodecyl sulfate
TCA	Trichloroacetic acid
TMRM	Tetramethylrhodamine methyl ester
Δψm	Mitochondrial membrane potential
