# Supplementary material for: A2A Receptor Activation Restores Lipid and Mitochondrial Homeostasis, Limiting Mycobacterium leprae Persistence in Human Monocytes
Source: Metabolites. 2026 Apr 29;16(5):304. doi: 10.3390/metabo16050304 (PMC13208476; doi:10.3390/metabo16050304)

Supplementary Figure S1: Treatment with adenosinergic pathway components does not affect monocyte viability. MTT assay showing cell viability of human monocytes under the indicated experimental conditions. Data are expressed as a percentage of control (untreated cells) and presented as mean ± SD from three independent experiments.

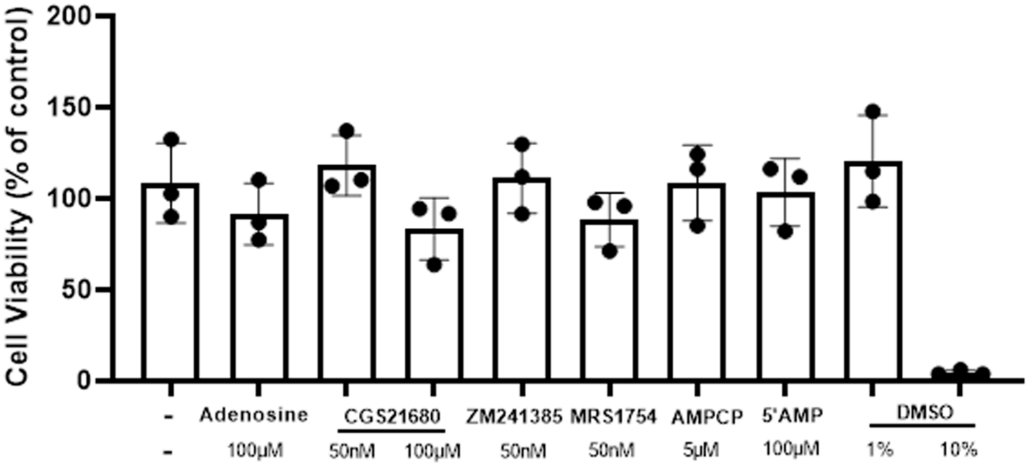

Supplement: Supplementary file 1 [file metabolites-16-00304-s001.zip › Supplementary Figure S1.pdf]
