# Supplementary material for: A2A Receptor Activation Restores Lipid and Mitochondrial Homeostasis, Limiting Mycobacterium leprae Persistence in Human Monocytes
Source: Metabolites. 2026 Apr 29;16(5):304. doi: 10.3390/metabo16050304 (PMC13208476; doi:10.3390/metabo16050304)

**Supplementary Figure S2: Dose–response analysis of CGS21680 on lipid droplet accumulation in *M. leprae*-infected human monocytes.** Enriched monocytes from PBMCs were infected with *M. leprae* (MOI 10:1) for 48h and treated with increasing concentrations of the $A_{2A}R$ agonist CGS21680 (50 nM, 1 μM, 10 μM, and 100 μM). (A) Representative fluorescence microscopy images showing lipid droplets (red) and nuclei (blue) under non-infected (NI) and infected conditions. Arrowheads indicate lipid droplets. Scale bar:10 μm. (B) Quantification of lipid droplet area per cell under the indicated experimental conditions. Data are presented as mean ± SD from at least three independent experiments conducted in duplicate. Statistical significance was determined by paired one-way ANOVA followed by Bonferroni's multiple comparisons test (*$P < 0.05$).

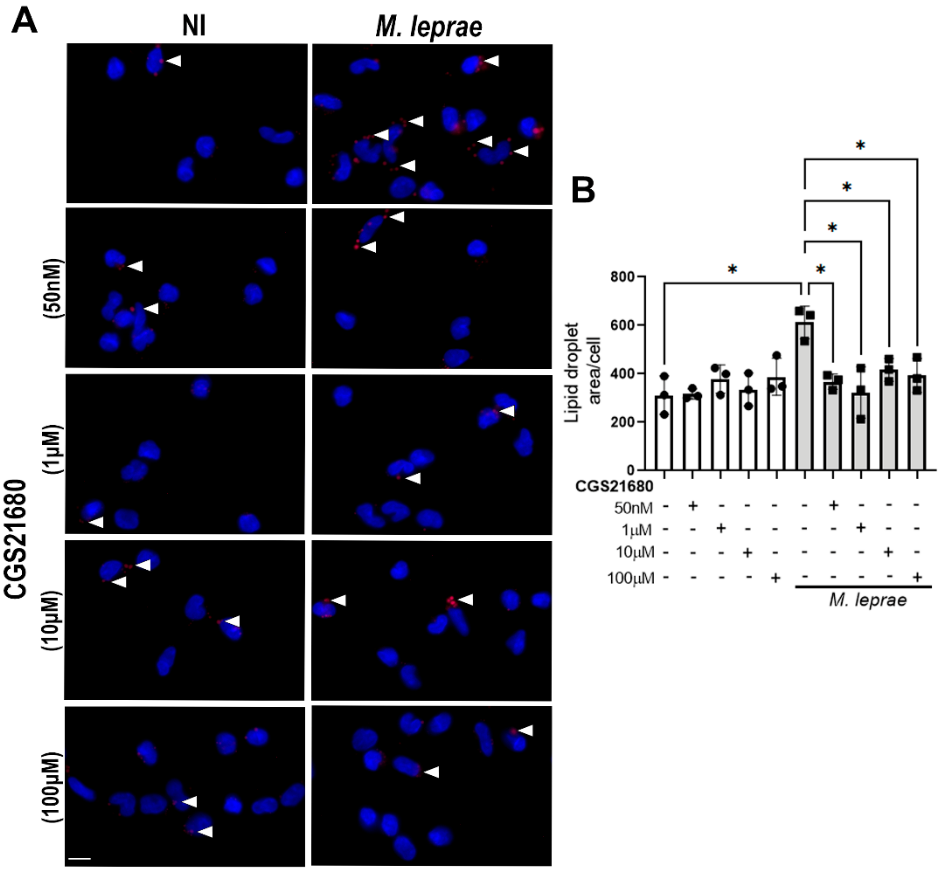

Supplement: Supplementary file 1 [file metabolites-16-00304-s001.zip › Supplementary Figure S2.pdf]
